# Solid-in-Oil-in-Water Emulsion: An Innovative Paradigm to Improve Drug Stability and Biological Activity

**DOI:** 10.1208/s12249-021-02074-y

**Published:** 2021-07-01

**Authors:** Anali Sawant, Seema Kamath, Hemanth KG, Girish Pai Kulyadi

**Affiliations:** Department of Pharmaceutics, Manipal College of Pharmaceutical Sciences, Manipal Academy of Higher Education, Manipal, Udupi, Karnataka 576104 India

**Keywords:** solid-in-oil-in-water emulsions, water-in-oil-in-water, solid-state proteins, entrapment efficiency

## Abstract

**Abstract:**

An emulsion is a biphasic dosage form comprising of dispersed phase containing droplets that are uniformly distributed into a surrounding liquid which forms the continuous phase. An emulsifier is added at the interface of two immiscible liquids to stabilize the thermodynamically unstable emulsion. Various types of emulsions such as water-in-oil (w-o), oil-in-water (o-w), microemulsions, and multiple emulsions are used for delivering certain drugs in the body. Water (aqueous) phase is commonly used for encapsulating proteins and several other drugs in water-in-oil-in-water (w-o-w) emulsion technique. But this method has posed certain problems such as decreased stability, burst release, and low entrapment efficiency. Thus, a novel “solid-in-oil-in-water” (s-o-w) emulsion system was developed for formulating certain drugs, probiotics, proteins, antibodies, and tannins to overcome these issues. In this method, the active ingredient is encapsulated as a solid and added to an oil phase, which formed a solid-oil dispersion. This dispersion was then mixed with water to form a continuous phase for enhancing the drug absorption. This article focuses on the various studies done to investigate the effectiveness of formulations prepared as solid-oil-water emulsions in comparison to conventional water-oil-water emulsions. A summary of the results obtained in each study is presented in this article. The s-o-w emulsion technique may become beneficial in near future as it has shown to improve the stability and efficacy of the entrapped active ingredient.

**Graphical abstract:**

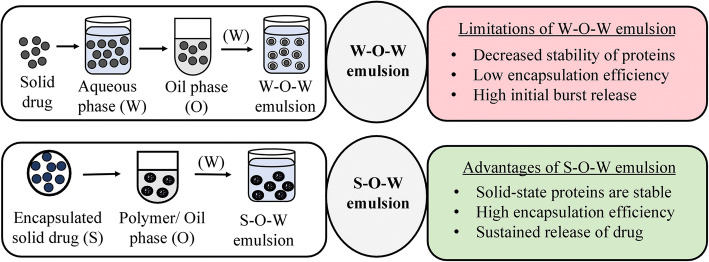

## INTRODUCTION

**Fig. 1:**
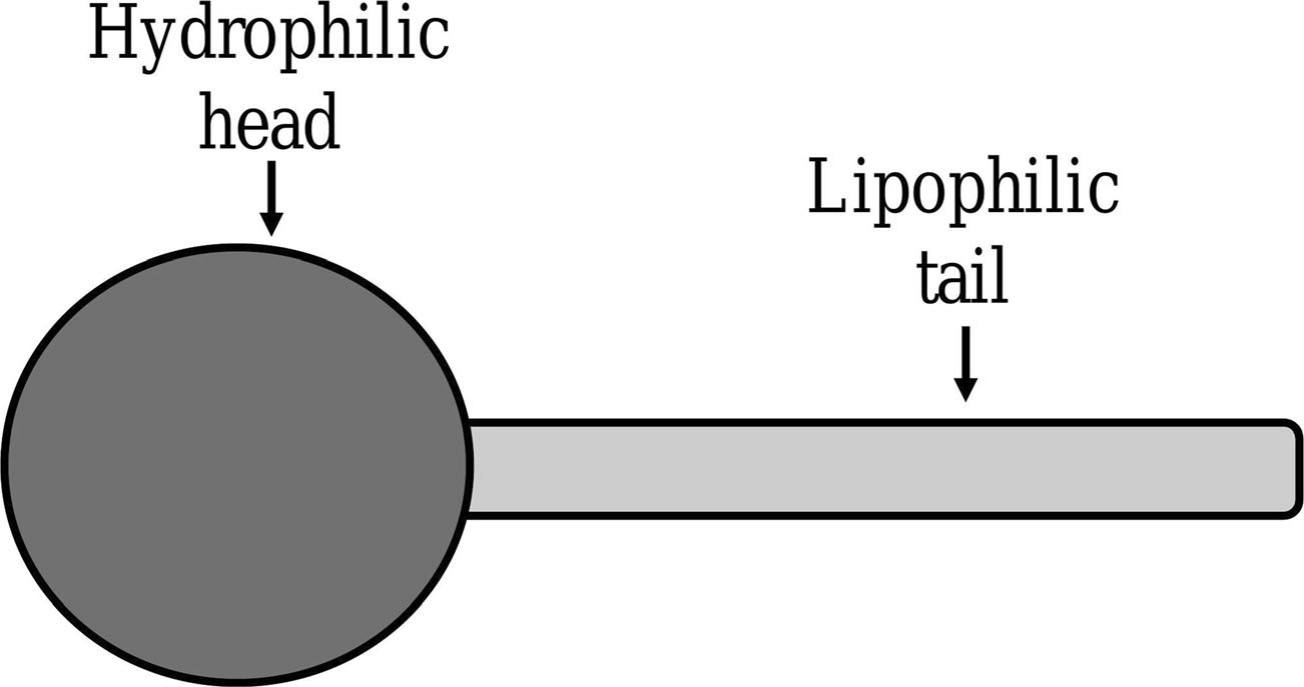
Emulsifier

**Fig. 2:**
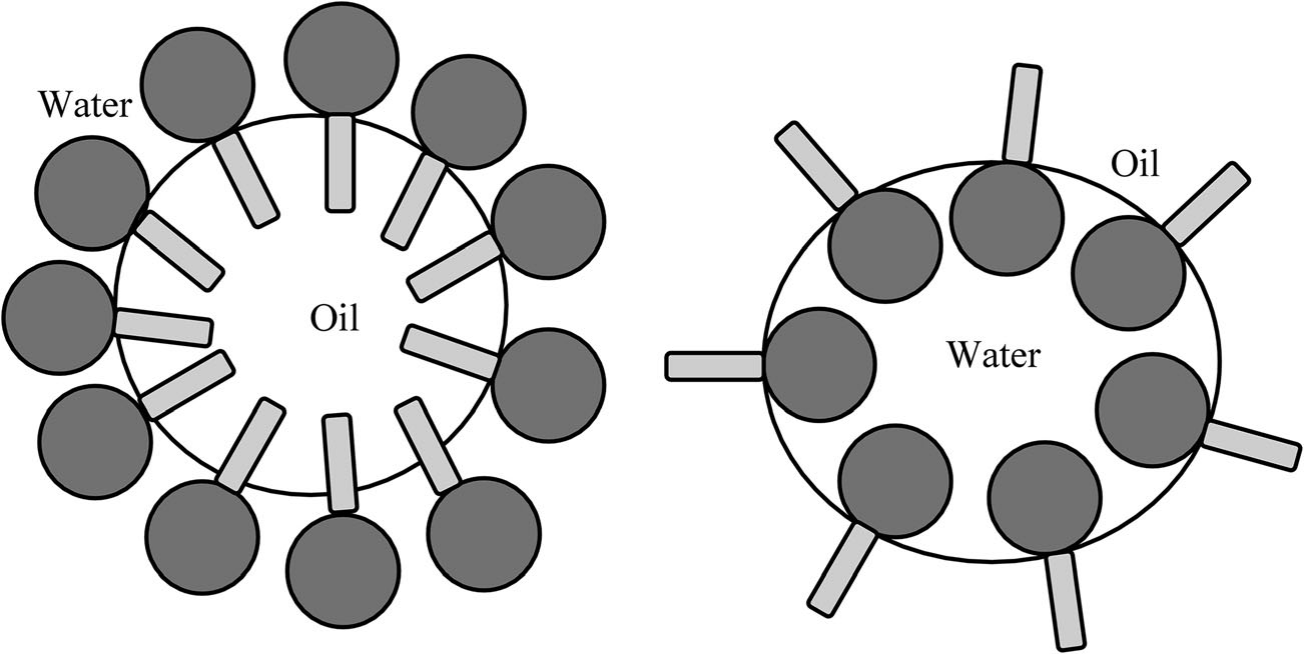
Arrangement of emulsifier

An emulsion is a subtype of colloid having a biphasic system containing two immiscible liquids, one is the dispersed particles (droplets) which is finely and uniformly distributed as globules throughout the second phase, the continuous phase (surrounding liquid) [1–3]. The average dispersed droplet diameter can vary from 100 nm to 100 μm [2]. Emulsions are added with emulsifiers (surfactants) at the point of interaction (boundary) of the two immiscible liquids as they are thermodynamically unstable. Hydrophilic−lipophilic balance (HLB) (suggested notably by Griffin) represents the oil and water solubility and indicates the class of emulsifiers. A certain type of emulsifier is added to each emulsion in order to stabilize it based on its HLB value. It also provides a way of selecting a suitable surfactant for a definite application. The emulsifiers can be described as amphiphilic as they contain both hydrophilic and hydrophobic parts (Fig. 1). Emulsifiers with HLB values 8–14 favor oil-in-water (o-w) emulsions and HLB values 3–6 favor water-in-oil (w-o) emulsions (Fig. 2) [4–6].

## TYPES OF EMULSIONS

### Water-in-Oil Emulsion

A w-o emulsion has a continuous phase formed of hydrophobic materials (oil) and water (globules) that make up the dispersed phase [1, 7]. Fingas and Fieldhouse discovered that when crude oil is combined with water, four definite w-o types of emulsions were developed: stable, meso-stable, unstable, and entrained emulsions. Stable w-o emulsions contain 60 to 80% of water on first day of preparation and are reddish-brown in appearance. Stable emulsions maintain stability for at least 4 weeks under laboratory conditions. The average properties of oil required to form a stable emulsion are as follows: density 0.9 g/ml; viscosity 300 mPa^*.*^s; resin content 9%; asphaltene content 5%; and asphaltene-to-resin ratio of 0.6. Meso-stable w-o emulsions are formed of brown or black viscous liquids with an average water content of 60–65% on first day of formation and less than 30% 1 week later and their properties lie between stable and unstable emulsions. The average properties of oil required to form a meso-stable emulsion are as follows: density 0.9 g/ml; viscosity 1300 mPa^*.*^s; resin content 16%; asphaltene content 8%; and asphaltene-to-resin ratio of 0.5. Unstable emulsions rapidly separate into two phases that are water and oil in a short time. Some water (usually less than about 10%) may be retained by the oil, especially if the oil is viscous. Entrained w-o types appear as black viscous liquids. It has an average water content of 40–50% on the first day of formation and less than 28% 1 week later. This type of w-o emulsion has a short life span. The average properties of oil required to form entrained w-o are as follows: density 0.97 g/ml; viscosity 60,000 mPa^*.*^s; resin content 18%; asphaltene content 12%; and asphaltene-to-resin ratio of 0.75. From these groups, only stable and meso-stable will be characterized as emulsions [8, 9]. w-o emulsion can easily release oil-soluble drugs as oil forms the continuous phase. This is favored for preparations used externally such as creams: example—cold cream [1].

### Oil-in-Water Emulsions

**Fig. 3:**
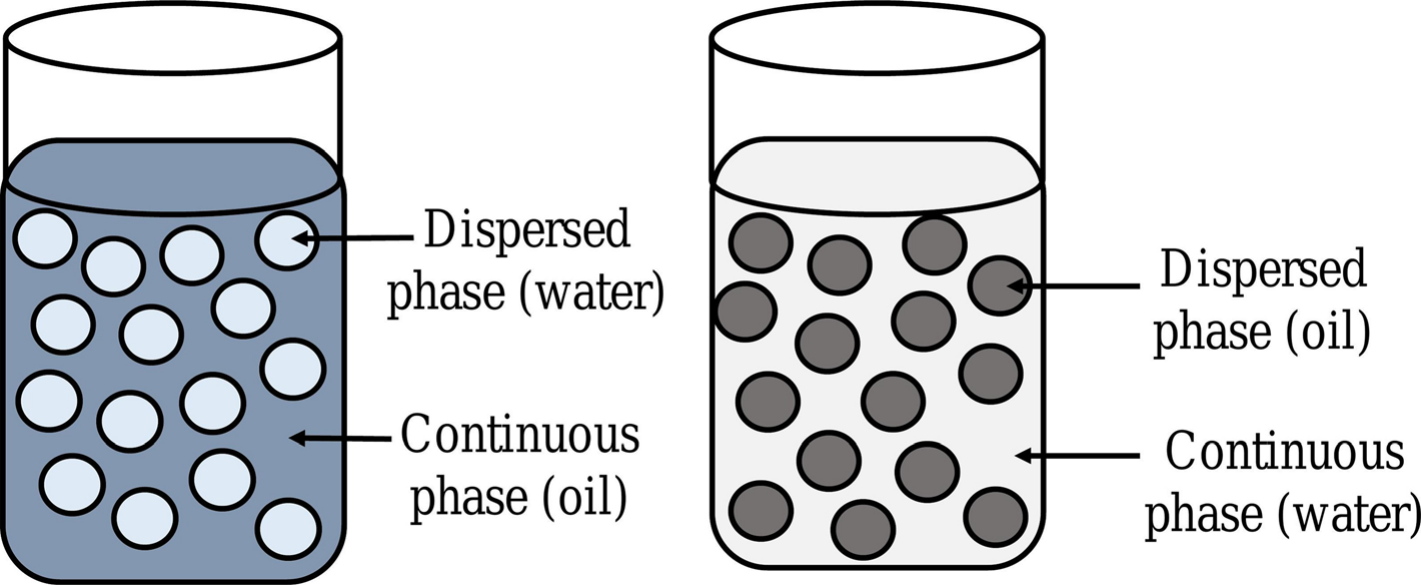
Water-in-oil and oil-in-water emulsion

In an oil-in-water (o-w) emulsion, the dispersed phase has oil droplets and the dispersion medium is an aqueous phase [1, 7]. It can be easily removed from the skin surface. They can be used internally to overcome the bitter taste of oil as well as used externally to give a cooling effect. As a result, lipophilic compounds (vitamins and antioxidants) can be delivered by using an o-w emulsions [10]. As water forms the external phase, water-soluble drugs can be easily released from such an o-w emulsion [1]. The w-o emulsions are frequently employed for delivering drugs as compared to o-w emulsions; therefore, o-w emulsions are often referred to as “reverse emulsions” (Fig. 3) [7].

### Nanoemulsions

Nanoemulsions have a droplet size of 10–100nm and are clear or translucent, thermodynamically stable, isotropic formulations made up of aqueous and oil phase added with emulsifying agents [11, 12]. They are formulated for delivering drugs because they are simple to prepare due to their feature of spontaneous emulsification, increased encapsulation of drug particles, and have stability for longer period of time [13]. They are categorized as w-o, o-w type and bi-continuous systems [14]. They enhance the bioavailability and solubility of bioactive food complexes and lipophilic formulations as they are lipid-based preparations [15].

### Microemulsions

**Fig. 4:**
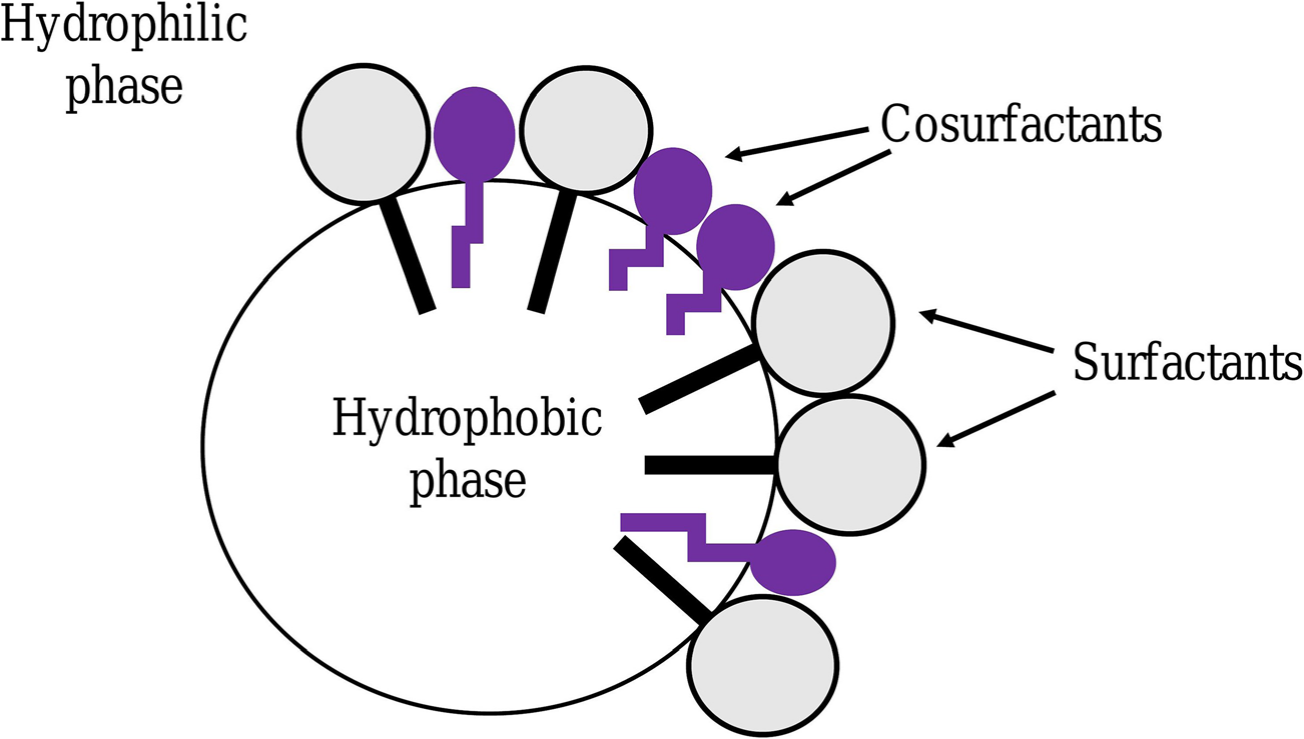
Microemulsion

Hoar and Schulman (1943) described microemulsions (MEs) as bicontinuous systems that consists of water, oil, and a surfactant added at the interfacial film frequently in combination with a co-surfactant (due to low-interfacial energy) forming a thermodynamically stable, clear dispersions with the ability to deliver hydrophobic and hydrophilic drugs into the skin (Fig. 4) [1, 16–19]. The two types of microemulsion are o-w and w-o microemulsions. Most appropriate formulation is oil-water (o-w) microemulsion, which enhances solubility by dispersing drugs with low water solubility into an oil phase of the emulsion. MEs can increase oral bioavailability by decreasing the droplet size (< 100 nm) and thus improve the rate of absorption due to surfactant-induced permeability changes [1, 20]. The key differences between emulsions and microemulsions are the structure, stability, and the droplet size of the dispersed phase [21].

Nanoemulsions and MEs majorly differ in their thermodynamic stability: nanoemulsion are thermodynamically unstable as the free energy of the colloidal dispersion (droplets in water) is higher than the free energy of the separate phases (oil and water), whereas MEs are thermodynamically stable as the free energy of the colloidal dispersion is lower than the free energy of the separate phases. Typically, a greater surfactant-to-oil ratio is required to prepare a microemulsion than a nanoemulsion. Only small molecule surfactants can be used to prepare MEs because only they are capable of generating ultralow interfacial tensions at particular monolayer curvatures. Whereas, small molecule surfactants, proteins and polysaccharides can be used as surface active agents to form nanoemulsion. Nanoemulsions tend to contain spherical particles, whereas MEs may form either spherical or non-spherical particles because of the ultralow interfacial tension [22]. Nanoemulsion generally have higher preparation cost and require mechanical shear for formation, whereas MEs have lower cost of preparation and can form by self-assembly. The concentration of surfactant is generally high (20% by weight) in microemulsion as compared to nanoemulsions (3–10% by weight) [23].

### Multiple Emulsions

**Fig. 5:**
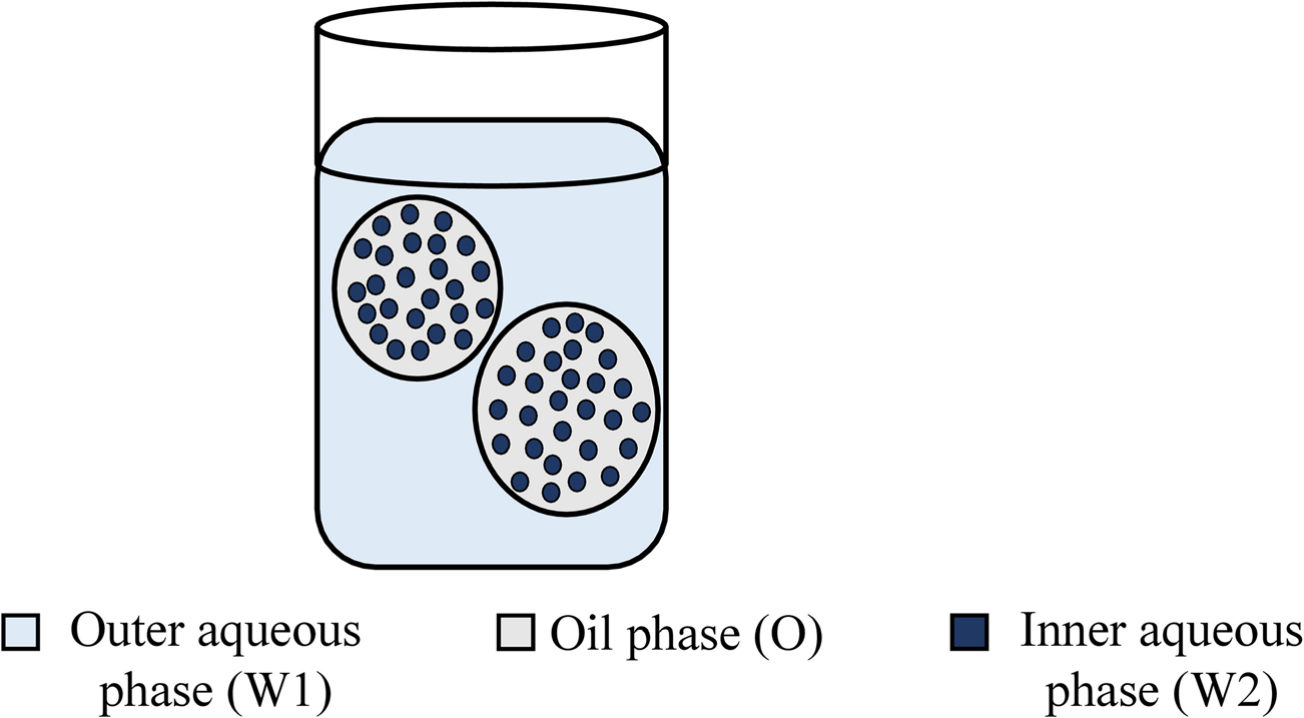
Multiemulsion W1-O-W2

These emulsions are complex polydisperse systems and can be considered as “emulsions of emulsion” or “double” or “triple emulsions” (Fig. 5). It is a system in which the o-w or w-o emulsions are dispersed in another liquid medium to form (water-oil)-in-water (w-o-w) emulsion or an (oil-water)-in-oil (o-w-o) emulsion [1, 2, 24]. Generally, a combination of hydrophilic and hydrophobic surfactants are added to stabilize multiple emulsions, one emulsifier having lower HLB value and another one having a higher HLB value are added [7, 25]. The major issue linked with multiple emulsions is thermodynamic instability and complex structure, which has reduced their effectiveness. Also, they are relatively complex to make, bulky, and susceptible to several routes of physical degradation [24, 26].

### Solid-in-Oil-in-Water Emulsion

**Fig. 6:**
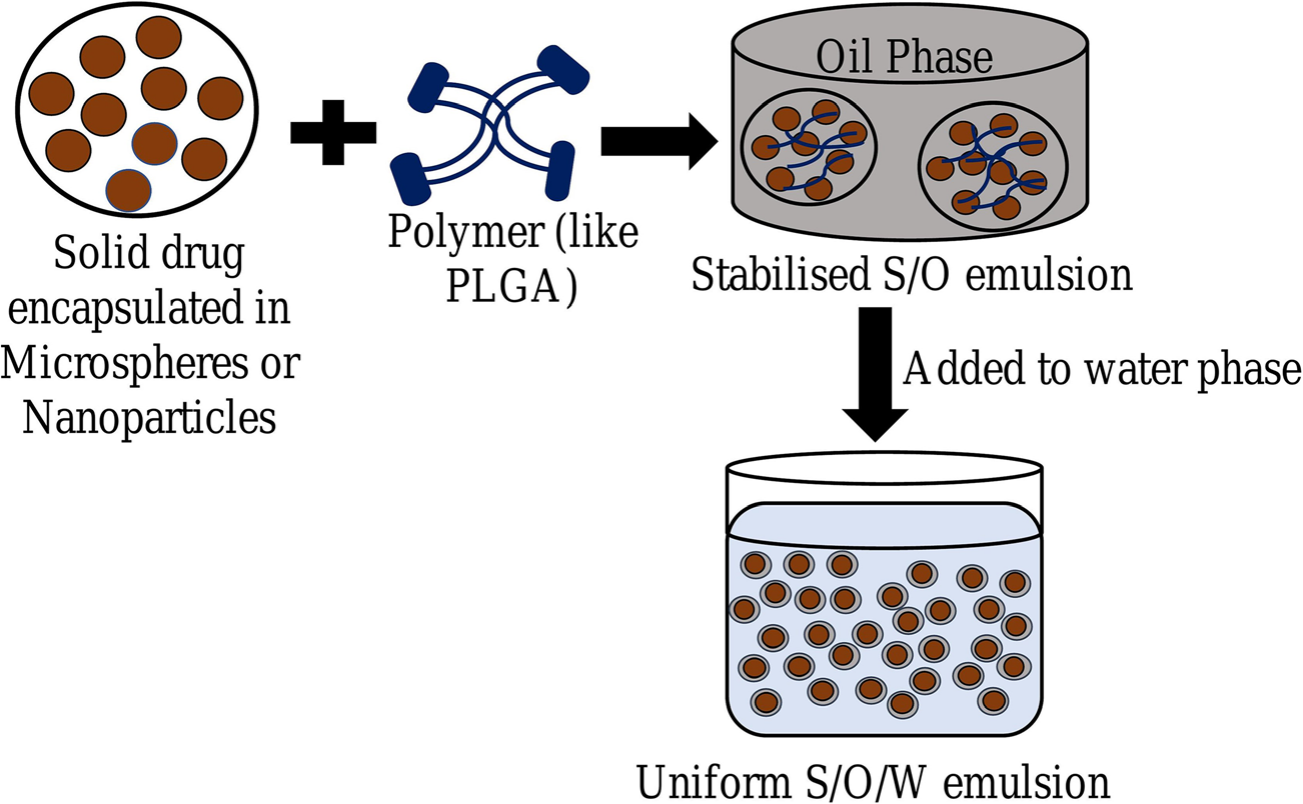
Solid-in-oil-in-water emulsion

For entrapping solid particles, drug may be dissolved in an oil phase to form a solid-oil dispersion followed by addition to the water phase, resulting in the development of a second emulsion. This entire process is known as the “solid-in-oil-in-water (s-o-w) double-emulsion method” (Fig. 6) [27]. The w-o-w emulsion dissolves protein in water and the aqueous phase of the emulsion entraps these hydrophilic drugs. The s-o-w emulsion technique is used to overcome certain difficulties (such as stability, encapsulation efficiency, burst release, agitation stress) generally encountered while entrapping hydrophilic drugs by w-o-w emulsion system [28–31]. Surface morphology studies have revealed that s-o-w emulsion particles exhibit smooth and spherical surfaces as compared to w-o-w emulsion [32–35]. Various factors contribute to stability of s-o-w emulsion; adsorption of solid particles at fluid interface that improves interfacial-film formation results in increased stabilization [16, 36]. Increased conformational mobility can lead to aggregation and unfolding. But solid-state proteins decrease conformational mobility and sustain their bioactivity as compared to large structural changes that arise when particles are in solution form [37, 38]. Moreover, a dissolution step is involved in s-o-w emulsion to dissolve solid-drugs which can decrease the drug loss from the organic phase leading to higher encapsulation efficiency and stability [30, 39]. The solid state of hydrophilic drug can greatly aid in preventing drug leakage caused due to anionic surfactant [32]. This method can effectively entrap sensitive materials with lesser damage and higher entrapment efficiency [40–42].

The studies elucidated below compare s-o-w emulsion and w-o-w emulsion techniques.

#### Novel s-o-w Emulsion Technique for IgG Encapsulation

Marquette *et al.* developed a s-o-w emulsion technique for preventing the denaturation of encapsulated protein that mainly arises in w-o-w emulsion system. The stability of IgG was maintained by encapsulating it in s-o-w emulsion by using a s-o-w evaporation/extraction technique [43, 44]. At room temperature, PLGA (poly D, L lactic co-glycolic acid) was added to ethyl acetate under magnetic stirring. Ethyl acetate was added to increase stability of IgG. A spray-dried form of IgG (SD IgG powder) was then added to an organic solution of PLGA to form the solid-oil (s-o) dispersion. This s-o dispersion was then blended with an external aqueous phase containing surfactant (PVA or PVP). The formed s-o-w emulsion was then added to a continuous phase of water to form the final s-o-w emulsion. The evaporation of ethyl acetate was achieved by magnetic stirring, which was further extracted. The microspheres were filtered, washed, and vacuumed at room temperature. The s-o-w emulsion method showed encapsulation efficiency of 60% (w/w) and drug loading of 6% (w/w) and it also preserved the integrity of the antibody [28, 45]. The stability of the IgG incorporated into the microspheres was evaluated by SEC-HPLC during the dissolution test and by quantifying amount of drug loaded. The effect of PLGA concentration on IgG stability during encapsulation process and release was statistically significant which stated that higher PLGA concentrations caused faster hardening of microspheres which may have a protective effect on the entrapped IgG. Moreover, high volume of external phase led to fast precipitation of the polymer contributing to faster hardening of microspheres. This fast precipitation contributed to an increase in the EE%, the drug loading, and the stability of the IgG inside the microspheres [28].

#### Microspheres Containing Diclofenac Sodium

Diclofenac sodium (DS) is a potent nonsteroidal anti-inflammatory drug (NSAID) (used in inflammatory and painful conditions) having low aqueous solubility and gastrolesive actions. Pediatric patients that undergo adenoidectomy and tonsillectomy are treated with DS which acts as a pain reliever (analgesic) [46, 47]. DS is a well-tolerated NSAID but side effects like perforation on the intestinal wall, bleeding, or ulcerations restrict its usage. [48, 49]. Oz *et al.* used the following two methods for preparation of the DS suspension. One was the conventional w-o-w emulsion method and other was the novel s-o-w emulsion method. DS-loaded microspheres were prepared using the novel s-o-w method. In this technique, Eudragit RS-100 was added to DCM (dichloromethane) forming the DCM solution, then DS (solid phase) was blended with the pre-formed DCM solution leading to the formation of a dispersion (s-o). A 5% NaCl solution or PVA made up the external water phase to which the above formed s-o dispersion was added directing the formation of final s-o-w emulsion. The microspheres were centrifuged, rinsed thoroughly, lyophilized, and then stored until further use. The results showed that both the formulations had high entrapment efficiency, but the percent drug loading capacity and amount of diclofenac in microspheres developed by s-o-w method were higher (13.01% and 150mg, respectively) as compared to w-o-w method (1.46% and 14 mg, respectively). The maximum pediatric daily dose of diclofenac is 75 to 150mg and the w-o-w emulsion microspheres could encapsulate only about 11.9mg of DS which is very insufficient. On the other hand, 88.40–100.0% of yield was seen in s-o-w microspheres prepared by the solvent evaporation method. Chemical stability of DS was confirmed using FTIR spectra. FTIR peaks of microspheres were almost unchanged suggesting no bond formation or chemical interaction between drug and polymer. This suggests that s-o-w emulsion system is advantageous for entrapment of diclofenac [50].

#### Theophylline Entrapped by s-o-w Method

PLGA has two α-hydroxy acids (lactic and glycolic acids) and is used to encapsulate peptides, antigens (proteins), lipopeptides, and plasmid DNA as it has an excellent safety profile in human and is approved by FDA for tissue engineering, vaccines, and drug delivery [51, 52]. Researchers have proposed two mechanisms by which the w_1_-o-w_2_ method causes the leakage of drug in the external aqueous phase from the internal aqueous phase (from w_1_ to w_2_) [53, 54]. The first mechanism states that drug can travel through the surfactant layer and the other is that the drug can diffuse across the oil layer by incorporating in the reverse micelles. As a result, s-o-w emulsion was developed by Toorisaka *et al.* In this s-o-w emulsion, first w-o emulsion was made by combining a homogenizer with an aqueous solution of hexane and hydrophilic drug (theophylline) containing hydrophobic surfactant (sucrose palmitate). A similar method was followed for coating the drug with the surfactant. s-o suspension was developed by dispersing surfactant-coated drug into methylene chloride. Next, PLGA was mixed with the s-o dispersion. Thus, the oil phase comprised of the above solution and the hydrophilic surfactant formed the aqueous phase. The mixing of these two phases results in a s-o-w emulsion. Centrifugation, drying, and lyophilization were done to collect the theophylline-PLGA particles. The w-o-w emulsion of theophylline had an encapsulation efficiency of 19.7%, but it dramatically improved in s-o-w emulsion (56.3%). Stability of the preparation was maintained by using 5% of lipophilic surfactant. In s-o-w emulsion, PLGA acted as a hydrophilic carrier for protein drugs and the size of oil droplets was controlled by membrane emulsification method [55].

#### Doxorubicin Microspheres for TACE Procedure

For patients with multinodular HCC (Hepatocellular carcinoma) and relatively preserved liver function, TACE (transcatheter arterial chemoembolization) is the recommended standard of care [56]. A hydrophilic drug can be delivered using a w-o emulsion of Lipiodol (iodized oil from poppy seeds), but incomplete embolic effect and immediate release are some of the disadvantages associated with this method [57, 58]. Doxorubicin (DOX; anthracycline drug) is usually used as an anticancer drug for pediatric and breast cancers, multiple myeloma, and Hodgkin’s and non-Hodgkin’s lymphoma [59]. It is challenging to efficiently encapsulate aqueous soluble drug (anticancer-DOX) in microspheres made up of PLGA, a hydrophobic polymer matrix. Thus, Choi *et al.* used s-o-w emulsification technique (solid drug added in oil layer) to increase the entrapment efficiency, avoid drug diffusion and drug leakage, and provide stability to the hydrophilic proteins. Doxorubicin was added to dimethyl sulfoxide (DMSO) followed by PLGA dissolved in acetone was added. Nitrogen gas stream was used to remove organic solvents. Next, the drug-polymer interface was added with DCM for dissolving the PLGA and suspending DOX, which forms s-o dispersion. This organic phase was then combined with PVA solution (2% w/v) and the resulting s-o-w emulsion was homogenized. When compared with o-w method, the drug encapsulation efficiency in the PLGA microspheres (s-o-w) was significantly higher (50.25%). PLGA microspheres showed a narrow size distribution with an average diameter of 26.36 ± 6.39 m. Thus, PLGA microspheres loaded with DOX (s-o-w method) can be used in TACE (liver tumor) for an efficient drug delivery [33].

#### TACE Procedure Using Miriplatin-Lipoidal Emulsion

The unequal sizes of anticancer drug-Lipiodol emulsion particles might cause insufficient outcomes of TACE using Lipiodol and thus, local tumor control can be achieved by a monodisperse antitumor agent-Lipiodol emulsion obtained by passing the formed emulsion through a SPG membrane [60]. Miriplatin, an analog of oxaliplatin (containing myristates as leaving groups and diaminocyclohexane (DACH) as a carrier ligand), is a lipophilic platinum derivative, specifically curated to have an improved affinity to Lipiodol and thus can easily disperse in Lipiodol to form a suspension leading to a gradual release of platinum [61]. Yasui *et al.* did a comparison of new s-o-w emulsion system and regular w-o-w emulsion system to check miriplatin distribution in tumors and to analyze the safety in Japanese white rabbits. Miriplatin powder was mixed with Lipiodol to form miriplatin suspension. Emulsion was developed by passing the suspension through a SPG membrane into an aqueous external phase. The surfactant used in this experiment was PEG 60 hydrogenated castor oil. A blend of HCO 60 and NaCl (0.45% w/v) made up the outer hydrophilic phase. The s-o-w emulsion had better Lipiodol accumulation in VX2 tumors in comparison to traditional system (no statistical difference) which may reflect the stable nature of the emulsion. While performing TACE, a sufficient margin is necessary for controlling tumor and to lower the local tumor recurrence [62] which can be achieved by s-o-w emulsion. The s-o-w emulsions may not lead to blockage of proximal arteries due to large droplets of Lipiodol avoiding the ischemic injury. Another major complication associated with TACE is ischemic injury of the bile duct although this type of injury was not seen in this study. This shows that s-o-w emulsion can become an alternative to the w-o-w method for the delivery of miriplatin [63].

#### ACTP-Loaded PLGA Microspheres

ACTP (altered collagen type II peptide; AP268-270) can effectively suppress the activation of T cell amongst other altered CII261-273 peptides and it can efficiently decrease joint injuries and joint inflammation in the CIA (collagen-induced arthritis) rat model [64, 65]. A s-o-w emulsion method was used by He *et al.* to incorporate ACTP in PLGA microspheres and to make ACTP, a more useful medication for rheumatoid arthritis. Primary s-o suspension was formed by dispersing lyophilized ACTP powder in PLGA solution (in methylene chloride) and the primary w-o emulsion was developed with an aqueous ACTP peptide solution added into PLGA solution (in methylene chloride). Then the final s-o-w emulsion was formed by mixing the s-o suspension and the w-o emulsion with an aqueous PVA solution (0.5% w/v) and NaCl (5.0%). A magnetic stirrer was used for evaporating the solvent. The formed microspheres underwent filtration followed by collection, rinsing with water, vacuum drying, and finally storing at −20°C. Loose and porous internal structure was seen in w-o-w microspheres, whereas a compact interior was seen in s-o-w microspheres. Microspheres prepared by conventional w-o-w technique had an encapsulation efficiency of 22.0–39.3%, whereas the encapsulation efficiency of PLGA microspheres prepared by novel s-o-w technique was significantly higher (69.7–79.8%). The initial burst in the s-o-w formulation was reduced considerably as the external aqueous phase was added with NaCl (5%). s-o-w microspheres showed higher peptide release rate. Thus, ACTP-loaded PLGA microspheres may be beneficial for treating rheumatoid arthritis [30].

#### An Insulin s-o-w Emulsion

It is tedious to control the size of particles in w-o-w emulsion as the inner w-o droplets are quite larger and it was noted that insulin lost activity at the interface when it was packed in w-o-w emulsion. But, the proteins maintained their activity when solubilized as solid-state in s-o-w emulsions [66–68]. In the novel s-o-w method prepared by Toorisaka *et al.*, the stable w-o emulsion was made by mixing hexane solution having ER-290, the lipophilic surfactant, and insulin aqueous solution. The insulin coated by surfactant was added to soyabean oil and dispersed using ultrasonication. A mixture containing soyabean oil with insulin and aqueous solution (comprising d-glucose, sodium cholate, and hydrophilic surfactant L-1695) was homogenized. Uniform particle size was achieved by passing the s-o-w emulsion through SPG membrane. The s-o-w emulsion proved advantageous as it showed no breakdown and coalescence of droplets and for 30 days a uniform diameter was retained. The effect after oral administration of s-o-w emulsion was evaluated in diabetic male Wistar rats. The hypoglycemic effect shown by s-o-w emulsion was prevalent for a long time and thus showed enhanced absorption of insulin. In this study, it was considered that Ostwald ripening effect [69] can be suppressed by emulsions that have a sharp particle size distribution causing increased stability for a long period of time. s-o-w emulsion formulated by a hydrophilic SPG membrane displayed a sharp size distribution and thus was stable. As a result, s-o-w emulsions are expected to be used in the treatment of diabetes [70].

#### PLGA Microspheres Loaded with Amoxicillin

Amoxicillin (structurally like ampicillin but differs only in the hydroxylation of the phenyl side chain) is a semi-synthetic drug belonging to the class of antibiotics called penicillin which provides a higher concentration in serum as it is well absorbed following oral administration. Also, greater excretion of amoxicillin is observed in urine in comparison to ampicillin [71, 72]. In this study, Xu *et al.* used PLGA to encapsulate amoxicillin (AMX), a hydrophilic drug and non-ionic as well as anionic surfactants were used to formulate microspheres using w-o-w and s-o-w emulsion techniques. The organic phase containing PLGA was mixed with AMX powder. The complete evaporation of dichloromethane was achieved by rapidly adding the dispersion to SDS solutions under vigorous stirring. AMX was entrapped in PLGA-microspheres by using DSS (1%) and PVA (1%) solutions, which formed the aqueous phase. The PLGA microspheres were centrifuged, washed, and lyophilized overnight followed by storage at 48°C. Table I compares the results of various parameters obtained in w-o-w and s-o-w emulsions.

**Table I:** Results Obtained in s-o-w and w-o-w Emulsion Systems [32]

Sample	System	AMX	Surfactant	Particle size	EE (%)
PLGA (s)	s-o-w	50 mg	DSS (1%)	4.7 ± 0.2 μm	37.4
PLGA (s)	s-o-w	50 mg	SDS (1%)	16.4 ± 0.8 μm	40.6
PLGA (s)	s-o-w	50 mg	PVA (1%)	23.4 ± 1.3 μm	61.0
PLGA (w)	w-o-w	0.2 mL (20%)	DSS (1%)	46.6 ± 4.5 μm	5.3
PLGA (w)	w-o-w	0.2 mL (20%)	SDS (1%)	40.2 ± 5.2 μm	4.0
PLGA (w)	w-o-w	0.2 mL (20%)	PVA (1%)	51.9 ± 3.8 μm	35.3

There was a significant reduction in particle size in s-o-w emulsion system (DSS and SDS, the anionic surfactants formed smaller microspheres as compared to PVA) than w-o-w emulsion method. When DSS (1%), SDS (1%), and PVA (1%) were used as surfactants, w-o-w system had less encapsulation efficiency (EE%) in comparison to s-o-w system. Highest EE% was seen when s-o-w emulsion was added with 1% PVA surfactant. The s-o-w microspheres containing PLGA along with surfactants were spherical having smooth surfaces and uniform size. Anionic surfactant may cause drug leakage and this effect was prevented by the solid state of the hydrophilic drug. It was observed by *in vitro* studies that PLGA microspheres in s-o-w system had an uninterrupted 30 days of release with an initial burst of 11% whereas microspheres in w-o-w system had a larger initial burst (45%) of amoxicillin within the first day. The surfactants did have an influence on the drug entrapment efficiency, but it did not influence drug release profiles greatly. Due to leakage of AMX and its electrostatic interactions, the emulsification ability of DSS and SDS is reduced making the w-o-w emulsion unstable. But in s-o-w emulsion, this effect was greatly prohibited by the solid state of drug making the emulsion stable. Proper selection of anionic surfactant is necessary for controlling the hydrophilic drug release from the negatively charged microspheres in s-o-w system [32].

#### Acacia Tannin Extract Encapsulated in Lipid Microparticles

Tannins (secondary metabolites of a plant) can tan or convert animal skin into leather. They can precipitate alkaloids, gelatins, or proteins and are thus gaining importance as nutraceuticals (functional foods, dietary supplements) but have certain limitations as astringency, bitter taste, and other consequences [73, 74]. Studies have demonstrated that encapsulation of bioactive extracts instead of raw products may help to reduce the limitations and improve bioavailability [75, 76]. The s-o-w method can offer advanced approaches for delivering anthocyanidins (bioactive substance) across GIT by controlling release of constituents in ingestion or digestion or by masking the flavors and odors [77, 78]. Adejoro *et al.* suspended ATE (*Acacia mearnsii* tannin extract) in a lipid solution of DCM containing a surfactant (Span 80) which forms the primary s-o phase. Tween 80 was blended in distilled water and the above formed mixture was added to it. This formed the s-o-w secondary mixture. The encapsulation of tannin was done based on the optimum requirements determined by the varying concentrations of ATE and aqueous phase. The mixture was magnetically stirred for the evaporation of DCM. The storage of microparticles was done at 4°C until further use. Extract of acacia tannin was used to avoid instability. The optimum amount of Tween 80 and Span 80 was decided based on preliminary tests. The parameters kept constant while preparing s-o-w microparticles were 0.1% w/v Tween 80 in water and 0.5% w/v Span80 in DCM, which maintained the stability of emulsion. The overview of s-o-w emulsion system results indicated that *in vitro* there was less methane and gas production, good morphological characteristics of microparticles, sustained release of tannin in microparticles of ATE encapsulated in lipid, and high entrapment efficiencies and thus this s-o-w technique can be used to regulate rumen fermentation [34].

#### Delivery of Lactase by s-o-w Emulsion Method

Bovine milk and dairy products have been widely used as human nutrition as it is a source of energy, minerals, vitamins, and fatty acids and contains lactose as an abundant solute that is digested by β-galactosidase (lactase) in the intestine [79, 80]. Lactose-intolerant individuals can have milk products by incorporating lactase in the formulation which hydrolyzes lactose to glucose and galactose [81]. Although lactase is obtained as an OTC medicine, it may get influenced by the proteases and pH of GIT and thus lose its activity *in vivo* [82]. Delivery of lactase can be achieved by s-o-w emulsions. Zhang *et al.* formed s-o dispersion by adding spray-dried liquid lactase into a solution of span 80 and milk fat (oil phase). The aqueous phase contained lecithin and whey protein isolate. s-o-w final emulsion was formed by mixing s-o dispersion and protein solution. Lactase is encapsulated in s-o-w emulsion, preventing its deactivation in simulated gastric fluid. During 14-day storage, all samples had no visible creaming, precipitation, or phase separation. Encapsulation enhanced thermal stability of lactase, probably due to exclusion of water molecules around the enzyme in s-o-w emulsions. Seventy-six percent of lactase powder was entrapped in s-o-w emulsion and therefore this system may be used for delivering lactase in milk especially in lactose-intolerant consumers [35].

#### Improvement in the Viability of Lactobacillus Salivarius

Probiotics are viable microorganisms that generate effects that are antagonistic to harmful bacteria and have useful biological effects on the host entity [83]. Lactobacillus salivarius, L. acidophilus, L. casei, and Escherichia coli are the common species used for making probiotics. The proteins generated by these lactic acid bacteria have antimicrobial activity [84, 85]. Probiotics are generally encapsulated to immobilize them and increase their survival rate in unfavorable conditions. A novel process of the s-o-w emulsion was used by Zhang *et al.* which encapsulated L. salivarius NRRL B-30514. The mixture comprised of a polymeric surfactant, sugar beet pectin (SBP), and solid core made of spray-dried cells and oil phase as soybean oil. s-o suspension was prepared by adding free L. salivarius to soyabean oil. It was further homogenized in SBP solution. In one more group of emulsions, calcium chloride (to crosslink SBP) was added after entrapping L. salivarius. The results indicated that emulsion with o:w ratio of 1:8 and SBP (3%w/v) showed a highest entrapment efficiency of 87%, and droplets were smaller than 17μm and thus SBP can be used to encapsulate L. salivarius using s-o-w emulsion method. Improved viability during storage and pasteurization, *in vitro* digestions seen in s-o-w emulsion due to entrapment of L. salivarius, proved to be advantageous. Divalent calcium ions were used to cross-link SBP which stabilized the oil droplets and further improved L. salivarius viability. This s-o-w emulsion can thus improve distribution of probiotic bacteria in food to enhance its viability [86].

#### Lecithin-Nanoparticles Loaded with Exenatide

Diabetes mellitus type 2 (T2DM) is indicated by dysfunction of β-cells of the pancreas and the target organs become resistant to insulin leading to insulin deficiency. This problem can be corrected by using incretins like DPP-IV (dipeptidyl peptidase-4) inhibitors that inhibit the lysis of GIP (gastric inhibitory polypeptide) and endogenous GLP-1 (glucagon-like peptide-1) analogs that have improved half-life and thus help in lowering blood glucose [87, 88]. The mechanism of GLP-1 receptor agonists is that it stimulates insulin and suppresses glucagon secretion in an approach that is reliant on glucose levels in the body. EMA and FDA approved the first incretin mimetic name exenatide which is used to treat T2DM and has similarities to mammalian GLP-1 [89]. The physical properties of two emulsion namely traditional w-o-w emulsion and modified s-o-w multi emulsion containing lecithin nanoparticles loaded with exenatide entrapped in PLGA microspheres (Ex-NPs) were compared by Dong *et al.* The oil phase was made of a solution containing acetone, PLGA, and DCM (o) whereas the solid phase comprised of Ex-NPs (s). This mixture was then added to a PVA solution (water phase; w) to form the s-o-w multiple emulsion. Microspheres were formed by evaporating the organic solvent by adding the sodium chloride to the emulsion contained in a flask. Centrifugation, rinsing, freezing, and lyophilization of microspheres was done and then they were collected. The results showed that s-o-w microspheres had reduced initial burst and sustained drug release for 60 days *in vitro*, higher entrapment efficiency, uniform particle size, and higher drug loading capacity (DLC) as compared to w-o-w microspheres. FTIR spectroscopy was used to investigate interactions between drug molecules and PLGA polymers. All the typical bands of Ex and PLGA were observed, indicating no interactions between Ex and PLGA. Characteristic bands of Ex were present in all the groups with their positions remaining consistent, suggesting that the molecular weight and structural integrity of Ex were not changed during the preparation of microspheres via both s-o-w and w-o-w methods. *In vivo* characteristics were better in s-o-w emulsion. Exenatide was released for 4 weeks in Ex-NPs-PLGA microspheres (s-o-w emulsion) when a single injection was given *in vivo* by subcutaneous route. Therefore, microspheres developed by s-o-w technique may be used for treating type 2 diabetes [90].

#### An *In situ* s-o-w Emulsion Containing Insulin

Polar organic solvents (such as DMSO) can help conserve the biological activity of proteins having low molecular weight and peptides because they are easily soluble in it and they are also devoid of advanced three-dimensional structures. DMSO can be used up to 50mg residual solvent/day and no justification is needed as it is a “solvent with low toxicity potential lyophilized” as prescribed by ICH Q3C guideline [91]. As a result, Bao *et al.* prepared microspheres using a novel *in situ* s-o-w process. Swine insulin was dissolved in DMSO and this solution was added to DCM containing PLGA (50:50) and the mixture was emulsified. The final s-o-w emulsion was developed by adding the formed emulsion to an aqueous solution of PVA. The organic solution was evaporated and the microspheres were collected after centrifugation followed by washing and lyophilization. For the emulsion to have anti-solvent effect, the V_DCM_/V_DMSO_ must be larger. Encapsulation efficiency was 90% w/w or nearly 100% w/w when V_DCM_/V_DMSO_ > 1 with drug loading of 15% w/w and when V_DCM_/V_DMSO_ < 1 the encapsulation efficiency was found to be lower. This suggest that in s-o-w method, it is very critical to maintain the ratio of the volume of DCM to DMSO solution (V_DCM_/V_DMSO_). The conventional w-o-w microspheres had a higher initial burst in comparison to microspheres formulated by *in situ* s-o-w technique as a sustained release of drug is achieved by compact inner structure expanding to the surface which helps in the formation of a stable emulsion [92].

#### PLGA Microspheres Containing Pramipexole-HGNS for Parkinson’s Disease

Pramipexole (PRX) used for treating Parkinson’s disease (PD) is a specific agonist of dopamine (D2) receptor. It is given as an adjunct with levodopa or as monotherapy and was permitted for use in PD by FDA in 1997 [93, 94]. Tremor at rest, rigidity, akinesia, and postural instability (TRAP) are key attributes of PD (“shaking palsy” by Dr. James Parkinson) [95, 96]. MPTP (1-methyl-4-phenyl-1,2,3,6-tetrahydropyridine) is a neurotoxin that causes degeneration of dopamine neurons [97]. Untimely, repeated and rigorous dosage schedule is essential for PRX due to small half-life, which restricts its usage [98]. Li *et al.* used near infrared (NIR) light for on-demand drug release and PLGA microspheres loaded with HGNS (hollow gold nanospheres) and PRX were formulated. Two methods namely s-o-w and w-o-w methods were developed. The oil phase of s-o-w emulsion had various ratios of PLGA contained in DCM and PRX contained in DMSO. This phase was added with HGNS. This dispersion was mixed with PVA (1% w/v). The formed emulsion was suspended in more volume of PVA (0.1% w/v) and hardened to make microspheres. Microspheres were cleaned, centrifuged, added to adequate quantity of water, lyophilized, and preserved at 4°C. Rapid drug release was modulated by NIR laser as photothermal effectiveness was powerful in s-o-w emulsion (both in *vivo* and *in vitro*). The optimum drug release profile of s-o-w system facilitated the Sprague-Dawley (SD) rat’s neurons to quickly retrieve from damage caused by MPTP as exhibited by immunohistochemistry and pharmacodynamic studies. TEM analysis of HGNS morphology revealed a near-spherical shape and uniform size distribution without apparent aggregation indicating a stable emulsion [99].

Generally, methylene chloride (dichloromethane) is used as organic phase and PVA (polyvinyl alcohol) is used as aqueous phase in the formulation of s-o-w emulsion [100–105]**.**

*Table* *II* summarizes additional studies related to s-o-w emulsion from published literature.

**Table II:** Summary of Outcomes of s-o-w Emulsion and their Stability

Therapeutic agent	Polymer	Significant outcomes of s-o-w emulsion	Stability	References
Proteins				
Bovine serum albumin (BSA)	PEG-6000	• High yield of product (at least 70%) • Protein was highly pure, suggesting that this method will form quality products	4:1 ratio of PEG:BSA yielded extremely stable suspension	[106]
	PLGA	• Encapsulation efficiency (EE) was >90% with an encapsulation yield of 70% • Cumulative release of >90% with low initial burst.	BSA structure was less perturbed as measured by FTIR	[100]
	PLGA	• The EE was 76% in s-o-w emulsion • Good biocompatibility and biosafety without notable cytotoxicity	FTIR peaks showed no interactions within components	[107]
Recombinant human growth hormone (rhGH)	PLGA	• Stable plasma concentration • Bioactivity of rhGH was retained • Cumulative release >90% and burst <15% • Improved IGF level in blood and increased body weight in hypophysectomized rat model	Not reported	[101]
	PLGA	• EE was >90% when zinc oxide was added • Addition of zinc oxide caused higher serum levels following subcutaneous injection • *In vitro* profile revealed complete release of rhGH	Zinc stabilized rhGH within microcapsules	[102]
Human growth hormone (hGH)	PLGA-grafted dextran (Dex-g-PLGA) graft copolymers	• EE was >94% • Constant plasma level was maintained for a week • IGF-1 concentration increased gradually over 4 days indicating that biological activity of hGH was maintained	Not reported	[108]
Erythropoietin (EPO)	PLGA	• EPO native state was preserved • Prolonged efficacy in mice did not compromise anti-EPO antibodies development	Less aggregation was seen in s-o-w microspheres	[109]
	PLGA/PLA	• Sustained-release profile • Prolonged RGC survival in optic nerve crushed rats upon intraperitoneal injection	Not reported	[110]
Interleukin-18 (IL-18)	PLGA	• Solid form of protein decreases its interactions with PLGA • Amounts of active IL-18 and a subsequent discharge of 16.5 ± 8.4ng/day for 21 days was enough to validate *in vivo* therapeutic strategy	Protein integrity was preserved at 4°C during first 3 h	[111]
Drugs				
Ranibizumab	PLGA	• Drug retained 94% of its bioactivity • Sustained release profile with lesser burst release	Bands on SDS-PAGE showed no detectable fragments or aggregates	[104]
Bevacizumab	PLGA/PCADK	• *In vivo* profile showed increased bioavailability of bevacizumab • Highly tolerability in ocular tissue • Lack of chronic or transient IOP, thus safe for intravitreal injection	Addition of PCADK stabilized bevacizumab	[103]
Lornoxicam	PLGA	• Cumulative amount released over 32 days was > 80% • Enhanced drug targeting in joint cavity due to the prolonged retention of drug	Higher viscosity restricted migration of drug resulting in stability	[112]
Phenytoin sodium	Nanotubes with PLGA	• Controlled drug release in gastric medium • Reduced side effects and drug loss	More chemical stability was seen in acidic (gastric) medium	[105]
Tegafur and 5-fluorouracil (5-FU)	Tween 80	• Mean residence time was higher enabling sustained and prolonged release formulation	Not reported	[113]
Sulfasalazine and betamethasone	PCL, PLA, PLGA	• Vast difference in EE of sulfasalazine (11% in w-o-w and 73% in s-o-w) • 30% of the entrapped drugs were released after 12 h with less pronounced burst effect	Not reported	[39]
Melarsoprol	PCL	• High entrapment of drug (161 μm/mg) • Relatively prolonged release (about 50% in 2h) that reached 80% after 7h	XRD showed distinct peaks of PCL and melarsoprol	[114]
Disodium norcantharidate (DSNC)	PCL	• EE as high as 75% was obtained with drug loading of 25%. • *In vitro* profile displayed rapid release phase followed by slow-release phase	XRD indicated no bonding between components	[115]
Miscellaneous				
GDNF (glial cell line–derived neurotrophic factor)/vit E (vitamin E)	PLGA	• Significant increase in RGC survival in cultures treated with GDNF • GDNF/vit E combination was effective for at least eleven weeks after a single intravitreal injection in an animal model of glaucoma	Low-binding Eppendorf tubes maintained GDNF stability	[116]
γ-Chymotrypsin and horseradish peroxidase (type II)	PLGA	• Encapsulation prevented the inactivation of protein • Optimized formulation showed an EE of 76%	PEG stabilized γ-chymotrypsin and horseradish peroxidase in PLGA microspheres.	[117]
Glutamine	Milk fat	• 90% release in simulated intestinal fluid • Minimum release of bioactive compounds during storage and maximum gradual release in intestine	pH was kept optimum to maintain the stability of emulsion.	[118]
Low molecular weight heparin (LMWH)	PLGA	• Initial burst reduced (12.4%), EE (76.8%) and yield (84.5%) increased upon addition of 5% NaCl. • *In vitro* profile showed sustained release for about 14 days • Single injection elevated anti-factor Xa activity levels for about 6 days	FTIR results demonstrated that LMWH was stable after encapsulation	[119]
Hydroxyapatite (HAp)	PLGA	• Unique porous microspheres with dense core were formed which could be utilized for loading large molecules	PLGA stabilizes HAp	[120]

PEG polyethylene glycol, *IGF* insulin-like growth factor, *PLA* polylactic acid, *RGC* retinal ganglion cell, *PCADK* poly (cyclohexane-1,4-diyl acetone dimethylene ketal), *IOP* intraocular pressure, *PCL* polycaprolactone, *XRD* X-ray diffraction

## ADVANTAGES

- In s-o-w method, a complex of drug and surfactant diffuses in oil layer and therefore the emulsion particle size can be controlled and it can help in preventing the loss of aqueous soluble substances from the emulsions [55].
- It is a useful method to maintain protein integrity as solid-state proteins are stable in a hydrophobic solvent [29]
- “Water organic solvent interface” development is prevented in the solid-in-oil first emulsion [31].

## CHALLENGES

- Drugs can be easily micronized without loss of activity but micronization of protein particles remains a key issue with s-o-w emulsion as protein may denature, although this can be controlled by lyophilization or co-lyophilization [37, 42, 67].
- As oil/water interface still exists, entrapped particles can get dissolved or hydrated by penetrated water. The contact of therapeutic agent with oil/water interfaces should also be considered [121].
- Further studies need to be done to analyze the integrity of delicate materials encapsulated within the matrix of microspheres [121].
- As there are limited studies related to s-o-w emulsion, detailed investigation needs to be done to determine the correct weight of therapeutic agent and volume of oil, its ratio, type of polymer/copolymer, and/or surfactant along with its concentration so as to develop a compatible formulation with optimal properties [32, 38, 44, 50, 55].

## POTENTIAL OF FORMULATING S-O-W EMULSION SYSTEM

Solid system of drugs is favored (due to added benefits) in contrast to liquid form in a multiemulsion. Certain *in vivo* animal studies [63, 90, 99, 122] have demonstrated the benefits of replacing w-o-w emulsion with s-o-w emulsion system. Further, clinical studies can be done to verify the effectiveness of encapsulated drug inside the body.

## CONCLUSION

The results for the above-mentioned studies indicate that the s-o-w emulsion system can overcome limitations associated with the w-o-w emulsion system and thus prove to be a valuable method for encapsulating drugs and substances that are unstable or show reduced stability in w-o-w emulsion. Entrapment efficiency improved, lower initial burst release was seen, and the stability was enhanced in novel s-o-w emulsion. This technique proves to be advantageous because it encapsulates the drug in solid form which may have contributed to its stability in the formulation. As a result, this method can be further explored to entrap active ingredients and thus enhance its biological activity.
